# Measuring anterior trunk deformity in scoliosis: development of asymmetry parameters using surface topography (a pilot study)

**DOI:** 10.1186/s13013-016-0096-5

**Published:** 2016-10-14

**Authors:** Patrick Knott, Baron Lonner, Mark Smith, Erin Frommeyer, Yuan Ren

**Affiliations:** 1 Rosalind Franklin University of Medicine and Science, North Chicago, IL USA; 2 Mount Sinai Beth Israel, New York, NY USA; 3 Mount Sinai Hospital, New York, NY USA

**Keywords:** Surface Topography, Anterior Trunk Asymmetry, TAASQ

## Abstract

**Background:**

Clinicians who assess and treat patients for scoliosis typically use parameters that are all visible from the posterior view. Radiographs assess the internal spinal deformity, but do not directly evaluate body shape, either posterior or anterior. This is problematic, as the patient is most concerned about the way they appear in the mirror. An objective set of anterior measurements is needed to help quantify the anterior asymmetry that is present in scoliosis.

**Methods:**

The design of this system of assessment was developed as a consensus of thinking from four points of view. A spine surgeon provided the musculoskeletal structural perspective. A plastic surgeon specializing in breast reconstruction provided the aesthetic and soft tissue perspective. A surface topography researcher provided the imaging perspective, and a scoliosis patient provided the self-perception and emotional perspective.

Using an iterative process, a series of potential measurement parameters using surface topography measurements were considered, debated, and ultimately selected to be part of a system of measurement that provides an overall assessment of anterior trunk asymmetry.

**Results:**

An anterior surface topography scan in the relaxed, standing position was taken of the scoliosis patient. The computer provides a 3D topographical model that is used to complete measurements that can be combined to achieve an Anterior Aesthetic Deformity Score. Shoulder parameters, including shoulder height difference and shoulder slope difference, make up 40 % of the total score. Breast asymmetry, including nipple height difference and sternal notch-to-nipple distance, make up 30 % of the total score. Waist asymmetry makes up the final 30 % of the score, providing an objective and quantifiable measure of anterior trunk deformity.

**Conclusions:**

These measurements provide an objective, systematic evaluation of anterior trunk asymmetry that can be used in the assessment of patients with scoliosis. Clinical research should now be done to validate this system and show that it is reproducible in a variety of settings and patients.

## Background

Adolescent Idiopathic Scoliosis (AIS) can cause significant disability and deformity, which can greatly impact a patient’s body image, self-esteem, and social abilities [1, 2]. The issue of body image is especially difficult for adolescents who are just reaching or are in the midst of puberty, when their bodies undergo rapid and dramatic changes. Adolescent girls with scoliosis in particular have a greater likelihood of low self-esteem and low self-image than adolescent boys [3, 4]. The thoracic asymmetry seen in AIS also has a strong association with breast asymmetry, particularly in curves >10° [5]. In addition, breast asymmetry in size, volume and position has been clinically observed in a large percentage of female AIS patients [6]. This is not to say that the frontal image of males is unimportant. A previous study showed that male adolescents with scoliosis were 97 % more likely to worry that their body was developing abnormally than their normal male peers. Furthermore, in the same study 79 % of boys with scoliosis had greater concern about the quality of their peer relations and had poorer body image [7]. Regardless of gender, body image is intrinsically associated with quality of life.

The Truncal Anterior Asymmetry Scoliosis Questionnaire (TAASQ) is an instrument developed and validated to evaluate patient concerns related to self-perceived anterior trunk appearance, and how those concerns might affect patients’ psychological mindset and behavior [8, 9]. The TAASQ is also designed to better allow healthcare providers to attend to the needs and concerns of their patients with scoliosis. It focuses on concerns of breast asymmetry, hip and waist asymmetry, and rib anterior prominence.

It consists of 20 scoreable items that appraise:

1. Unease about body part(s) felt to be asymmetrical
2. Preoccupation with the concern(s)
3. Emotional distress or worry over self-perceived asymmetry
4. Behavioral modification due to the self-perceived asymmetry
5. Pain or impairment of function

Repetitive radiographic examination is a standard modality for assessing the progression in scoliosis. The increased risk of cancer is considerable in AIS patients, as childhood exposure to radiation imparts the highest risk of developing cancer later in life [10]. And while radiographs remain a necessity for treatment, there should still be an effort to limit exposure as much as possible. Furthermore, radiographs do not provide any direct information about the body shape of the individual afflicted with scoliosis. The Formetric Surface Topography system (DIERS Medical Systems, Inc. Chicago, IL) was developed to use harmless light to create a 3D reconstruction of the spine and trunk shape as opposed to the x-ray’s 2D depiction [11]. Surface topography has been utilized to assess the posterior deformity and changes associated with scoliosis over time as a radiation-free assessment tool [11–14]. However, there is lack of research currently available to quantify the anterior deformity caused by scoliosis, and how the anterior trunk deformity can affect self-perceived body image, quality of life and patients’ psychological mindset and behavior.

The aim of this project was to develop an objective anterior asymmetry scoring system using surface topography. This scoring system should quantify the amount of anterior asymmetry in a way that is useful to both the physician and the patient. Input from several perspectives needed to be obtained in the development of this model. A Delphi Method of collaboration was chosen as the best way to proceed.

## Methods

A team was developed that included a surface topography researcher, a scoliosis spine surgeon, a plastic surgeon specializing in breast reconstruction, and a female scoliosis patient. This group would bring the different perspectives necessary to create an objective scoring system that would have validity for the different groups of people who would use it.

Using the Delphi method of iterative collaboration and consensus, an initial proposal for an anterior trunk scoring system was developed, and then edited and modified over four iterations until a complete group consensus was reached. The level of agreement went from moderate in round one to high in round 4. During each iteration of editing, the score was used on several torso models to see how the scoring methods would result in a different score based on the influence that each parameter had on the total score.

## Results

The final scoring system is based on three major areas of measurement. The first is the shoulder asymmetry. This was given the most influence on the total score because it is the asymmetry that is most easy to notice on the scoliosis patient. Even when fully clothed and from a distance, an observer can tell when a patient has shoulders that are at different heights, and have different sloping lines going towards the base of the neck. In the topographical model, the slope of each shoulder from the base of the neck to the acromion is taken, and the difference between the two slopes is recorded as half of the shoulder score. The other half is taken from the slope of the line that connects the two acromion processes. The difference of this line from the horizontal is recorded. These two halves are equally weighted.

**Fig. 1:**
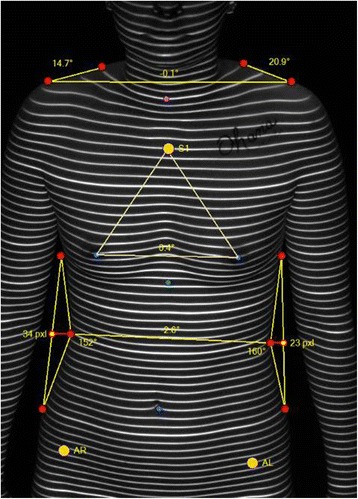
Anterior image with measurements in place

Figure 1 shows a right shoulder slope of 14.7°, and a left shoulder slope of 20.9°, for a difference of 6.2°. The line connecting the two acromion processes is 0.1°, or almost perfectly horizontal. Together, these two measurements make up 40 % of the total asymmetry score.

The breast asymmetry is first measured by the line that connects the two nipples, in comparison with the horizontal. This is recorded as an angular measurement and is measured as 0.4° in Fig. 1. Next, a measurement of the distance from the sternal notch (marked S1 in the figure) to each nipple is calculated, and the difference is recorded in millimeters. Finally, the rotation of the trunk in the axial plane at the level of the breasts is recorded by comparing the line connecting the two nipples to the line connecting the two Anterior Iliac Crests at the waist. These three measurements, equally weighted, together form the breast score, which makes up 30 % of the total asymmetry score.

And third, the waist asymmetry is measured by drawing a triangle along the right and left waistlines. The apex of the triangle should be located in the place where the waist curves inward the most. The other two points of each triangle should mark the waist at the spot where the breast meets the chest wall laterally, and at the level of the most lateral protruding part of the waist. The obtuse angle of each triangle is compared, and the difference between them is recorded in degrees. These are seen in Fig. 1 as 152° on the right, and 160° on the left. Next, the line that connects the apex of each triangle is compared to the horizontal, and this angle is recorded in degrees. It is seen as 2.6° in the figure. Finally, the depth of each triangle is measured, and in the figure these are seen as 34 and 23 pixels on the right and left. The difference between them is recorded as a percentage (eg. 23/34 = 0.68) These three measurements are equally weighted in the waist score, which makes up the final 30 % of the total asymmetry score.

## Discussion and conclusion

This formula for calculating anterior asymmetry must now be validated through further research. It will be compared to the TAASQ in adolescent patients with scoliosis to see whether the magnitude of the score reflects the visual assessment of deformity reached by the spine surgeon, the scoliosis patient, and the patient’s family.

The process by which this formula was reached, however, takes into account several important points of view in assessing trunk asymmetry. This is the first step in validating this as a useful tool for measurement.
